# A Short Synthesis of Vellosimine and Its Derivatives

**DOI:** 10.1021/acs.joc.3c00905

**Published:** 2023-06-02

**Authors:** Barbara Chatinovska, Rokas Gegevičius, Edvinas Orentas

**Affiliations:** Department of Organic Chemistry, Institute of Chemistry, Vilnius University, Naugarduko 24, LT-03225 Vilnius, Lithuania

## Abstract

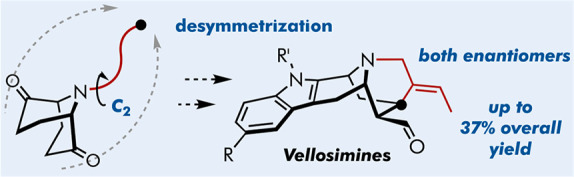

Rapid access to both enantiomers of vellosimine and its derivatives is secured from a readily affordable *C*_2_-symmetric 9-azabicyclo[3.3.1]nonane precursor available in both enantiomeric forms. The strategy reported leverages desymmetrization via intramolecular cyclization used to assemble the key intermediate with two differentiated carbonyl groups. Late-stage site selective indolization enables a concise synthesis of vellosimines and a straightforward diversification of the alkaloid scaffold.

Sarpagine monoterpene alkaloids represent a family of structurally related bioactive compounds featuring an indole unit fused with a diversly functionalized azapolycyclic molecular framework. Found in several plants belonging mainly to *Apocynaceae* and *Gelsemiaceae* families, these alkaloids possess diverse biological properties.^1−3^ Owing to their considerable biological activities and interesting chemical structure, vellosimine **1** and its derivatives, such as **2** and **3** (Figure 1a), still stimulate extensive interest and have been the subject of several recent total syntheses.^4^

**Figure 1:**
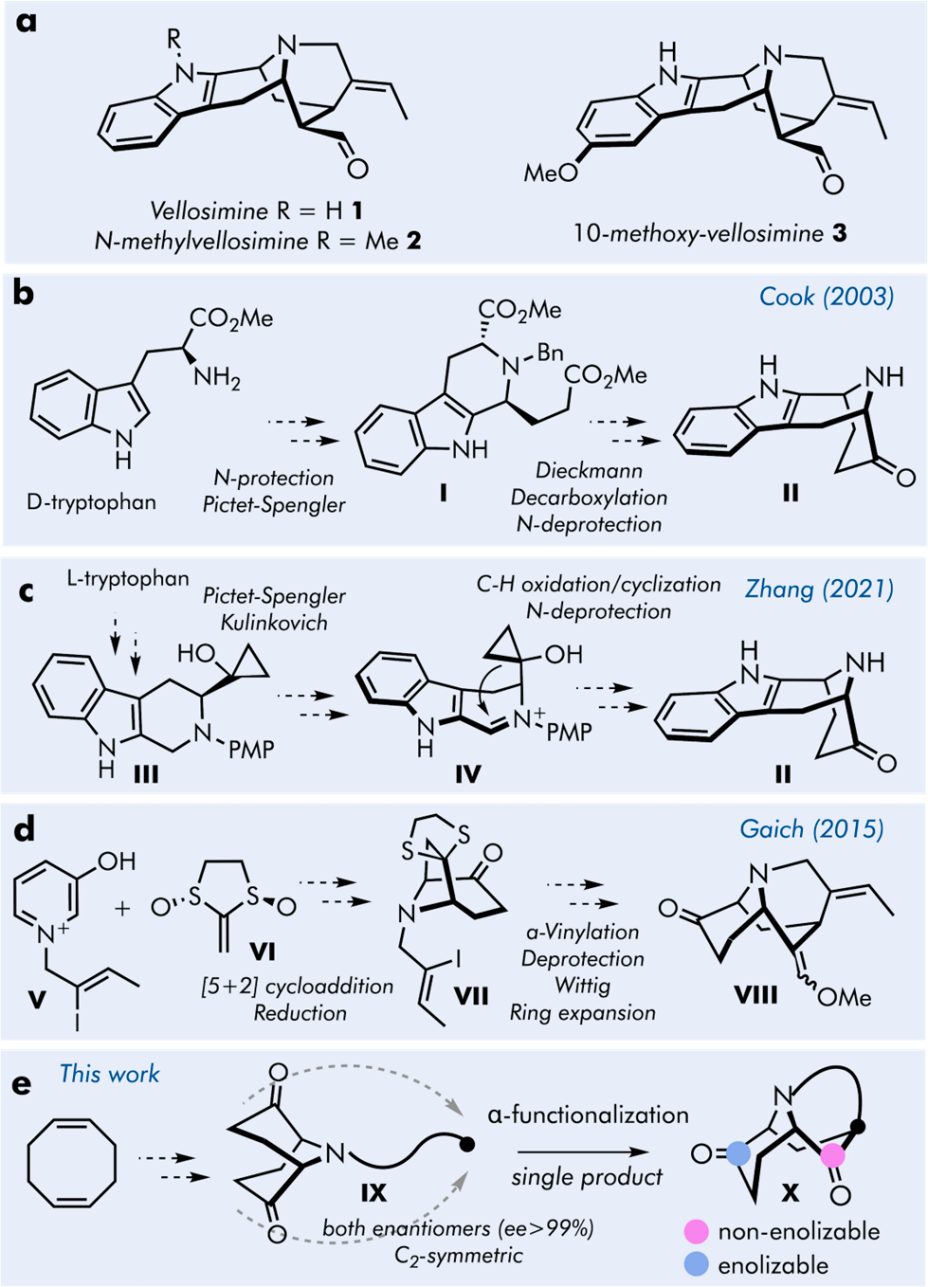
(a) Structures of vellosimine and its derivatives; prior art (b–d) and our desymmetrization approach toward vellosimine (e).

The pioneering studies toward the total synthesis of sarpagine alkaloids were conducted by Cook and co-workers (Figure 1b).^4a−4c^ Given the presence of the indole ring in the structure of **1**–**2**, the choice of tryptophan as a starting material, similar to the biosynthetic pathway, was well justified (Figure 1b). Moreover, the chirality of starting material was fully transferred during Pictet–Spengler cyclization to provide enantiopure intermediate **I**. On the other hand, unnatural d-tryptophan had to be used to establish the correct stereochemistry of the 9-azabicyclo[3.3.1]nonane scaffold produced in the ensuing Dieckmann condensation. Subsequent dealkoxycarboxylation afforded the key intermediate **II** which can be further elaborated to vellosimine in 3 steps.

Natural L-tryptophan served as a starting material for the most recent synthesis of **1** by the Zhang group (Figure 1c).^4g^ The cyclopropanated intermediate **III** was accessed in a few steps utilizing Pictet–Spengler and Kulinkovich reactions. The azabicyclo[3.3.1]nonane scaffold was then assembled by an elegant tandem C–H oxidation–cyclization reaction, where the cyclopropanol C–C bond acted as a nucleophile toward the iminium species. The tricyclic core of target alkaloids was forged by connecting the bridge nitrogen in **II** with the α-position of the ketone, but in this case enolate–alkyne cyclization was used instead of α-alkenylation, pursued by the Cook group.

Although the use of l- or d-tryptophan is advantageous in obtaining enantiomerically pure intermediates, the early stage introduction of an indole core complicates further diversification of products. To overcome this limitation, Gaich and co-workers proposed a different intermediate **VIII**, featuring the required azatricyclic framework equipped with a masked aldehyde in the form of vinyl ether and the free carbonyl group (Figure 1d).^4d−4f^ The latter was envisioned to serve as a handle for Fischer indolization to afford indole-substituted derivatives, not only the natural ones, such as **2** and **3**, but also non-natural congeners for biological screening. The route toward **VIII** encompassed the ring expansion of **VII** which in turn was obtained from an enantiospecific [5 + 2] cycloaddition reaction employing enantiopure **VI**.

In our report, we propose an alternative strategy to easily access a general-purpose intermediate **X** in both enantiomeric forms (Figure 1e). The approach is based on desymmetrization of *C*_2_-symmetric azabicyclic diketone **IX** possessing a reactive tether on a nitrogen atom. In this way, an intramolecular cyclization reaction would deliver a tricyclic product **X** having two differentiated carbonyl groups. The enolizable one is expected to readily engage in Fischer indolization reaction, whereas the nonenolizable carbonyl group should not react beyond the hydrazone step. Although two hydrogen atoms are located at α-positions, the latter carbonyl group is truly nonenolizable because of Bredt’s rule. Moreover, the formation of a hypothetical indoline-type product also seems unfeasible considering the large geometric constraint resulting from the nearly orthogonal positioning of the C=O (Figure 1e, purple label) double bond and bridgehead hydrogen atom.

Our synthesis commenced from a large scale preparation of a racemic *endo*, *endo*-*N*-benzyl-9-azabicyclo[3.3.1]nonane-2,6-diol **6** according to a procedure recently optimized in our laboratory (Scheme 1).^5,6^ Namely, the required *bis*-*syn* diepoxide **5** was obtained in 77% yield from cheap cyclooctadiene **4** using Oxone as oxidant to generate dimethyldioxirane epoxidation agent *in situ*. The reaction can easily be scaled up to 50 g, and the crude product obtained was pure enough to be used in the next step. *syn*-Diepoxide **5** was then converted to 9-azabicyclo[3.3.1]nonane diol **6** by the method of Rassat in nearly quantitative yield.^7^ The resolution of enantiomers of **6** was achieved by kinetic resolution using lipase from *Candida rugosa* (CRL) as previously described.^5^ Both enantiomers can be obtained in enantiopure (*ee* 99+%) form which opens the way for the synthesis of the unnatural antipodes with unexplored biological properties.

**Scheme 1:**
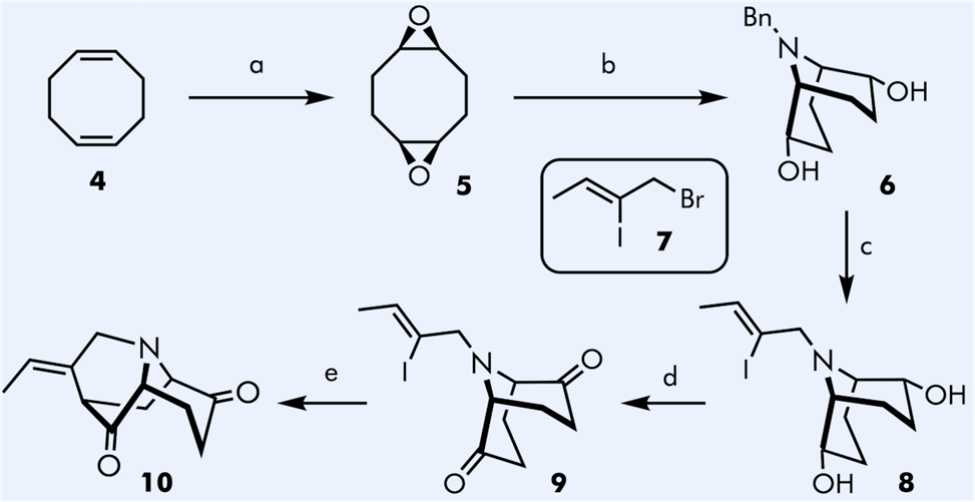
Synthesis of the Key Intermediate Reagents and conditions: (a) Oxone, NaHCO_3_, H_2_O–acetone–ethyl acetate, rt −30 °C, 1 h, 77%; (b) BnNH_2_, H_2_O, reflux, overnight; (CF_3_CO)_2_O, DCM, −60 °C; Et_3_N, DCM, reflux, overnight, one pot, 95%; (for (+)-**6**) *Candida rugosa*, vinyl acetate, rt, 46% (c) Pd/C, H_2_, MeOH, AcOH, rt, overnight; (*Z*)-1-bromo-2-iodobut-2-ene **7**, K_2_CO_3_, THF, reflux, 72 h, one pot, 89%; (d) (COCl)_2_, TEA, DMSO, DCM, −60 °C to rt, 90%; (e) PhOH, KO^t^Bu, Pd(PPh_3_)_4_, THF, reflux, 4 h, 73%.

After hydrogenative debenzylation of **6**, the secondary amine was directly alkylated with allylic bromide **7**(8) to afford diol **8** in 89% overall yield. Subsequent Swern oxidation provided diketone **9** in 90% yield. The desymmetrization of **9** was undertaken using palladium catalyzed α-vinylation following Bonjoch conditions (Pd(PPh_3_)_4_, PhOK)^9,4c^ to furnish the key intermediate **10** in 73% yield. The synthesis was also repeated using enantiopure (+)-(*1S*,*2S*,*5S*,*6S*)-**6** in essentially identical yields and delivered diketone **10** possessing opposite chirality to natural vellosimine.

With diketone **10** in hand, site selective Fischer indolization was investigated next (Table 1). Treatment of **10** with a slight excess of phenylhydrazine under neutral conditions resulted in the formation of the corresponding hydrazone of unknown regioselectivity.

**Table 1:**
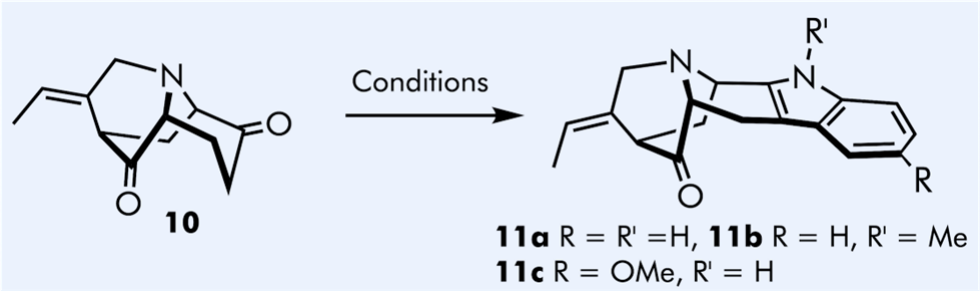
Optimization of Fischer Indolization Reaction^a^

entry	hydrazone formation	indolization	hydrazone cleavage	yield (%)
1	1.2 equiv PhNHNH_2,_ EtOH, reflux 1 h	2.5 M HCl/MeOH, reflux 6 h	1.0 equiv 2,4-DCBA, HCl, CHCl_3_, rt, overnight	trace
2	1.2 equiv PhNHNH_2,_ EtOH, reflux 2 h	2.5 M HCl/MeOH, reflux, overnight	1.1 equiv 2,4-DCBA, HCl, CHCl_3_, rt, overnight	trace
3	1.2 equiv PhNHNH_2_/2.5 M HCl/MeOH, reflux, overnight		1.1 equiv 2,4-DCBA, HCl, CHCl_3_, rt, overnight	trace
4	1.2 equiv PhNHNH_2_/glacial AcOH, reflux, overnight		1.1 equiv 2,4-DCBA, HCl, CHCl_3_, rt, overnight	–
5	2.5 equiv PhNHNH_2_/EtOH, reflux overnight	2.5 M HCl/MeOH, reflux, overnight	10 equiv DCBA, HCl/MeOH, rt, overnight	87
6	2.5 equiv PhNMeNH_2_/EtOH, reflux overnight	2.5 M HCl/MeOH, reflux, overnight	10 equiv DCBA, HCl/MeOH, rt, overnight	40^b^
7	2.5 equiv 4-OMe-PhNHNH_2_/EtOH, reflux overnight	2.5 M HCl/MeOH, reflux, overnight	10 equiv DCBA, HCl/MeOH, rt, overnight	61^b^

a DCBA = 2,4-dichlorobenzaldehyde.

b Racemic **10** was used.

The indolization reaction was then performed without purification of the intermediate hydrazone using acidic conditions. After the reaction, the crude was treated with 2,4-dichlorobenzaldehyde (DCBA) to release a nonenolizable carbonyl group via hydrazone exchange. A near-stoichiometric amount of phenylhydrazone provided only trace amounts (<10%) of the target compound **11** under various conditions tried (Table 1, entries 1–4). Most likely, the nonproductive hydrazone formation at the nonenolizable site was exclusively taking place. Gratifyingly, the simultaneous increase of both phenylhydrazine and hydrazine scavenger DCBA to 2.5 and 10 equiv, respectively, resulted in clean production of **11a** in 87% yield. If desired, the excess of DCBA can be recovered performing acid–base extraction. The same procedure was also successfully applied to 1-methyl-1-phenylhydrazine (Table 1, entry 6) and 4-methoxyphenyl hydrazine (Table 1, entry 7) to provide the corresponding indole derivatives.

Lastly, the obtained ketone **11a** was converted to vellosimine **1** in 72% yield, using a sequence of Wittig olefination and enol ether hydrolysis, performed without the purification of the intermediate enol ether (Scheme 2). The spectral properties of **1** were identical to those previously reported,^4d,4g^ except for the opposite optical rotation angle. Racemic vellosimines **2** and **3** were obtained in analogous fashion in 28% and 68% yields, respectively.

**Scheme 2:**
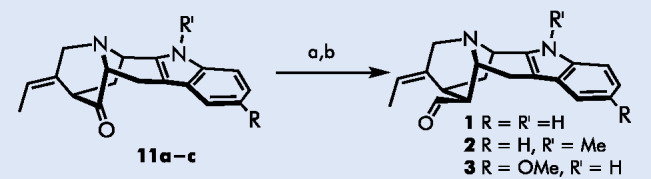
Completion of the Synthesis of Vellosimines Reagents and conditions: (a) MeOCH_2_PPh_3_^+^Cl^–^, KO^t^Bu, toluene, rt, overnight; (b) 2.0 M HCl, THF, 55 °C, overnight. Overall yields 72% (**1**), 28% (**2**), 68% (**3**).

In conclusion, the synthesis of (−)-vellosimine was successfully completed in overall 37% yield starting from available 9-azabicyclo[3.3.1]nonane precursor. The strategy disclosed herein, based on desymmetrization and differentiation of two carbonyl groups, not only provides the access to both enantiomers of vellosimine, but also enables the late stage diversification of the indole ring, as demonstrated with the synthesis of *N*-methylated and 10-OMe substituted vellosimines.

## Experimental Section

### General Procedures

All reagents used were purchased from commercial suppliers and used as provided. All the flasks used to carry out reactions were dried in an oven at 110 °C prior to use. Tetrahydrofuran (THF) was distilled over Na metal and benzophenone. Dichloromethane (DCM) was distilled over calcium hydride. All reactions were monitored by thin-layer chromatography (TLC) carried out on 0.25 mm Merck silica gel plates (60F-254) using either UV light (254 nm) for visualization or anisaldehyde in ethanol or 0.2% ninhydrin in ethanol as the developing agent and heat for visualization. Silica gel (Fluorochem, 40–63 μm) was used for flash column chromatography. ^1^H and ^13^C NMR spectra were recorded on an NMR spectrometer at 400 MHz for ^1^H and 101 MHz for ^13^C, respectively. ^1^H and ^13^C NMR spectra are referenced to residual solvent (CDCl_3_, 7.26 and 77.16 ppm for ^1^H NMR and ^13^C NMR, respectively). When necessary, assignments were obtained by reference to COSY, HSQC, and HMBC correlations. Chemical shifts are reported in ppm, and multiplicities are indicated by br (broad), s (singlet), d (doublet), t (triplet), q (quartet), m (multiplet), and combinations thereof. High-resolution mass spectra (HRMS) were recorded on an ESI/QTOF instrument. Optical rotations were measured on a KRÜSS P3001RS automatic digital polarimeter at 589 nm; [α]^D^_T_ values are given in 10^–1^ deg cm^2^ g^–1^, and concentrations are given in units of g/100 cm^3^.

Abbreviations: AcCl, acetyl chloride; DCM, dichloromethane; DMSO, dimethyl sulfoxide; EA, ethyl acetate; MeOH, methanol; PE, petrol ether (40–60 °C fraction); Py, pyridine; TEA, triethylamine.

#### (±)-9-((Z)-2-Iodobut-2-en-1-yl)-9-azabicyclo[3.3.1]nonane-2,6-diol (**8**)

Amine (±)-**6** (5.00 g, 20.0 mmol) was dissolved in MeOH (200 mL), and glacial acetic acid (3.5 mL, 60.0 mmol) was added. The mixture was bubbled through with a stream of argon, and Pd/C 10% (213 mg, 2.00 mmol) was added. The reaction flask was evacuated and backfilled with hydrogen. The cycle was repeated 3 times, and the reaction was stirred under a hydrogen atmosphere overnight at room temperature. Upon completion, the reaction mixture was filtered through a Celite pad, and the filtrate was evaporated under reduced pressure. The obtained clear oil was dissolved in THF (100 mL), and then (*Z*)-1-bromo-2-iodobut-2-ene **7** (6.26 g, 24.0 mmol, 1.2 equiv) and K_2_CO_3_ (5.53 g, 40 mmol, 2.0 equiv) were added. The reaction mixture was refluxed for 72 h (oil bath). The solvent was evaporated, and the crude product was purified by column chromatography using PE:EA 1:1 as the eluent to afford **8** as a white solid (5.98 g, 89% yield). HRMS (ESI^+^) calcd. for C_12_H_21_INO_2_ ([M + H]^+^) 338.06170, found 338.0608. ^1^H NMR (400 MHz, CDCl_3_) δ 5.85 (q, *J* = 6.4 Hz, 1H), 4.16–3.97 (m, 2H), 3.50 (s, 2H), 2.70 (t, *J* = 5.2 Hz, 2H), 2.04–1.90 (m, 4H), 1.88–1.82 (m, 1H), 1.79 (d, *J* = 6.4 Hz, 3H), 1.77–1.62 (m, 3H), 1.46 (s, 2H). ^13^C{^1^H} NMR (101 MHz, CDCl_3_) δ 130.94, 111.71, 68.40, 64.23, 54.71, 30.25, 21.81, 20.49.

#### (±)-9-((Z)-2-Iodobut-2-en-1-yl)-9-azabicyclo[3.3.1]nonane-2,6-dione (**9**)

Oxalyl chloride (5.08 g, 4.5 equiv, 3.5 mL) was added to dry DCM (90 mL) in a Schlenk tube. The solution was cooled to −60 °C, and freshly distilled DMSO (7.5 equiv, 5.0 mL) was added slowly. After 20 min of stirring, the reaction mixture was warmed to −40 °C, and diol **8** (2.93 g, 8.7 mmol) was added swiftly. After 1 h, dry TEA (10 equiv, 13 mL) was added to the reaction mixture. The mixture was left to warm to room temperature, then was washed with H_2_O, and extracted with DCM (5 × 100 mL). The organic layer was dried with anhydrous Na_2_SO_4_. The solvents were evaporated under reduced pressure, and the crude product was purified by column chromatography using PE:EA 3:1 as eluent to afford **9** as a yellow oil (2.67 g, 90%). HRMS (ESI^+^) calcd. for C_12_H_17_INO_2_ ([M + H]^+^) 334.0304, found 334.0293. ^1^H NMR (400 MHz, CDCl_3_) δ 5.90–5.84 (q, *J* = 6.4 Hz 1H), 3.42 (d, *J* = 4.6 Hz, 2H), 3.35 (d, *J* = 7.1 Hz, 2H), 2.78–2.65 (m, 2H), 2.50–2.36 (m, 4H), 2.06–1.97 (m, 2H), 1.79 (d, *J* = 6.3 Hz, 3H). ^13^C{^1^H} NMR (101 MHz, CDCl_3_) δ 211.96, 133.92, 107.48, 63.44, 62.55, 35.30, 24.57, 21.84.

#### (±)-(E)-3-Ethylidenetetrahydro-2H-2,6-methanoquinolizine-1,7(6H,8H)-dione

(**10**) Phenol (56.5 mg, 0.60 mmol) and KO^t^Bu (50.5 mg, 0.45 mmol) were dissolved in dry THF (2.0 mL). The resulting mixture was stirred for 30 min. A solution of **9** (100 mg, 0.30 mmol) in THF (8.0 mL) was added dropwise with a syringe. The mixture was bubbled through with a stream of argon. Pd(PPh_3_)_4_ (26.0 mg, 0.023 mmol) was added to the reaction mixture. The reaction mixture was refluxed for 4 h (oil bath), and the solvent was evaporated. The resulting crude product was purified by column chromatography using PE:EA 2:1 as eluent to afford **10** as an orange amorphous solid (45 mg, 73%). HRMS (ESI^+^) calcd. for C_12_H_16_NO_2_ ([M + H]^+^) 206.1181, found 206.1175. ^1^H NMR (400 MHz, CDCl_3_) δ 5.50 (q, *J* = 6.9 Hz, 1H), 3.70–3.54 (m, 3H), 3.50 (t, *J* = 3.0 Hz, 1H), 3.35 (d, *J* = 4.9 Hz, 1H), 2.44–2.40 (m, 1H), 2.40–2.34 (m, 3H), 2.14–2.00 (m, 1H), 1.95 (dt, *J* = 14.1, 3.9 Hz, 1H), 1.65 (dt, *J* = 6.9, 2.2 Hz, 3H). ^13^C{^1^H} NMR (101 MHz, CDCl_3_) δ 216.20, 206.97, 131.67, 121.67, 64.13, 63.55, 55.03, 44.52, 32.05, 29.90, 24.44, 12.75.

### General Procedure I: Fischer Indolization

Diketone **10** (40 mg, 0.195 mmol) was dissolved in dry EtOH (2.0 mL), and the corresponding phenylhydrazine (free base or hydrochloride salt) (2.5 equiv) was added. The reaction mixture was refluxed overnight (oil bath). The mixture was cooled to room temperature, and the solvent was removed under reduced pressure. The reaction mixture was redissolved in 2.5 mL of dry MeOH followed by the slow addition of AcCl (0.17 mL). The reaction mixture was refluxed overnight (oil bath) and cooled to room temperature, and 2,4-dichlorobenzaldehyde (341.3 mg, 1.95 mmol, 10 equiv) was added. The resulting mixture was stirred overnight at room temperature, then quenched with TEA, and evaporated under reduced pressure. The crude product was purified by column chromatography using CHCl_3_:MeOH mixtures as the eluent.

#### (Z)-9-Ethylidene-5,6,9,10,11a,12-hexahydro-6,10-methanoindolo [3,2-*b*]quinolizin-11(8H)-one

(**11a**).^4b^ Following general procedure I using phenylhydrazine hydrochloride (70.5 mg). Purification by flash column chromatography (CHCl_3_:MeOH 50:1 + 1% TEA) afforded **11a** (47 mg, 87% yield) as a white solid. HRMS (ESI+) calcd. for C_18_H_19_N_2_O ([M + H]^+^) 279.1497, found 279.1482. ^1^H NMR (400 MHz, CDCl_3_) δ 8.50 (s, 1H), 7.48 (d, *J* = 7.8 Hz, 1H), 7.30 (d, *J* = 8.0 Hz, 1H), 7.21–7.09 (m, 1H), 7.10 (t, *J* = 7.1 Hz, 1H), 5.50 (q, *J* = 7.1 Hz, 1H), 4.37 (d, *J* = 9.6 Hz, 1H), 3.85–3.74 (m, 1H), 3.71 (d, *J* = 5.9 Hz, 1H), 3.42 (dd, *J* = 4.3, 1.9 Hz, 1H), 3.34 (dd, *J* = 15.8, 1.6 Hz, 1H), 3.09 (dd, *J* = 15.7, 6.2 Hz, 1H), 2.70 (br s, 1H) 2.46 (ddd, *J* = 11.9, 9.7, 2.0 Hz, 1H), 2.21 (dt, *J* = 13.0, 3.3 Hz, 1H), 1.65 (dt, *J* = 7.1, 2.0 Hz, 3H).^13^C{^1^H} NMR (101 MHz, CDCl_3_) δ 215.71, 136.57, 135.77, 131.37, 126.94, 122.24, 121.74, 119.86, 118.67, 111.25, 105.53, 64.30, 55.26, 50.93, 44.56, 36.35, 22.48, 12.84.

#### (Z)-9-Ethylidene-5-methyl-5,6,9,10,11a,12-hexahydro-6,10-methanoindolo [3,2-*b*]quinolizin-11(8H)-one (**11b**)^4b^

Following general procedure I using 1-methyl-1-phenylhydrazine (59.5 mg, 57 μL). Purification by flash column chromatography (CHCl_3_:MeOH 100:1 → 50:1 + 1% TEA) afforded **11b** (23 mg, 40% yield) as a beige solid. HRMS (ESI^+^) calcd. for C_19_H_21_N_2_O ([M + H]^+^) 293.1653, found 293.1646. ^1^H NMR (400 MHz, CDCl_3_) δ 7.51 (d, *J* = 7.8 Hz, 1H), 7.27 (d, *J* = 5.1 Hz, 1H), 7.16 (dddd, *J* = 39.1, 7.9, 6.9, 1.2 Hz, 2H), 5.59–5.51 (m, 1H), 4.38 (dd, *J* = 9.6, 2.5 Hz, 1H), 3.99–3.79 (m, 2H), 3.63–3.60 (m, 1H), 3.59 (s, 3H), 3.41 (m, 1H), 3.32 (m, 1H), 3.01 (dd, *J* = 15.5, 6.3 Hz, 1H), 2.54 (m, 1H), 2.15 (m, 1H), 1.68 (dt, *J* = 7.1, 2.0 Hz, 3H). ^13^C{^1^H} NMR (101 MHz, CDCl_3_) δ 217.23, 137.71, 137.56, 132.36, 126.68, 121.51, 121.20, 119.23, 118.65, 108.88, 104.64, 64.29, 55.66, 49.81, 44.47, 35.98, 29.43, 22.65, 12.79.

#### (Z)-9-Ethylidene-2-methoxy-5,6,9,10,11a,12-hexahydro-6,10-methanoindolo [3,2-*b*]quinolizin-11(8H)-one (**11c**)^4c^

Following general procedure I using 4-methoxyphenylhydrazine hydrochloride (85.1 mg). Purification by flash column chromatography (CHCl_3_:MeOH 100:1 + 1% TEA) afforded **11c** (37.0 mg, 61% yield) as a white solid. *Note: It was not possible to fully remove all impurities due to limited stability of this intermediate during column purification. It was therefore used in the next step immediately*. HRMS (ESI^+^) calcd. for C_19_H_21_N_2_O_2_ ([M + H]^+^) 309.1603, found 309.1595. ^1^H NMR (400 MHz, CDCl_3_) δ 8.41 (d, *J* = 9.6 Hz, 1H), 6.95–6.89 (m, 2H), 6.76 (dd, *J* = 8.7, 2.5 Hz, 1H), 5.47 (q, *J* = 7.1 Hz, 1H), 3.82 (s, 3H), 3.73–3.65 (m, 1H), 3.61–3.54 (m, 1H), 3.47 (ddt, *J* = 16.3, 5.7, 2.3 Hz, 1H), 3.35–3.30 (m, 1H), 3.29–3.22 (m, 1H), 3.01–2.83 (m, 2H), 2.34–2.10 (m, 1H), 2.04 (dt, *J* = 13.0, 3.2 Hz, 1H), 1.66–1.58 (d, *J* = 7.3 Hz, 3H). ^13^C{^1^H} NMR (101 MHz, CDCl_3_) δ 217.09, 154.31, 137.38, 132.16, 131.48, 127.29, 121.09, 112.13, 111.99, 105.10, 100.49, 64.13, 55.92, 55.10, 50.42, 44.65, 36.72, 22.63, 12.77.

### General Procedure II: Wittig Reaction

(Methoxymethyl) triphenylphosphonium chloride (250 mg, 0.73 mmol, 7.3 equiv) and KO^t^Bu (112 mg, 1.0 mmol, 10 equiv) were stirred in dry toluene at room temperature. A solution of indole **11** (0.01 mmol) in dry THF (1.0 mL) was added to the dark red solution of the ylide. The reaction mixture was stirred overnight at room temperature. The reaction mixture was quenched with H_2_O (1.0 mL) and washed with ethyl acetate (3 × 5.0 mL). The organic layer was separated and dried with anhydrous Na_2_SO_4_. The solvents were evaporated under reduced pressure. The resulting crude enol ether was redissolved in 2.0 M HCl (1.5 mL) and THF (1.5 mL) and was stirred at 55 °C overnight (oil bath). The reaction mixture was quenched with 2.0 M NaOH solution and extracted with DCM (3 × 15 mL). The organic layer was separated and dried with anhydrous Na_2_SO_4_. The solvents were evaporated under reduced pressure, and the crude product was purified by column chromatography.

#### (±)-Vellosimine (±)-**1**.^4g^

Following general procedure II using **11a** as starting material. Purification by flash column chromatography (CHCl_3_:MeOH 50:1 + 1% TEA) afforded (±)-**1** (21.0 mg, 72% yield) as an off-white solid. HRMS (ESI^+^) calcd. for C_19_H_21_N_2_O ([M + H]^+^) 293.1653, found 293.1648. ^1^H NMR (400 MHz, CDCl_3_) δ 9.64 (s, 1H), 7.85 (s, 1H), 7.47 (d, *J* = 7.7 Hz, 1H), 7.32 (d, *J* = 8.1 Hz, 1H), 7.16 (td, *J* = 7.6, 1.3 Hz, 1H), 7.10 (td, *J* = 7.4, 1.2 Hz, 1H), 5.43–5.29 (m, 1H), 4.22–4.10 (m, 1H), 3.62 (ddt, *J* = 12.4, 10.5, 2.7 Hz, 3H), 3.20 (dt, *J* = 4.0, 1.9 Hz, 1H), 3.14 (ddd, *J* = 15.6, 5.2, 1.1 Hz, 1H), 2.60 (dd, *J* = 15.6, 1.5 Hz, 1H), 2.51 (dt, *J* = 7.6, 1.5 Hz, 1H), 2.07 (ddd, *J* = 12.2, 9.9, 2.0 Hz, 1H), 1.82 (ddd, *J* = 12.6, 4.2, 2.4 Hz, 1H), 1.61 (dt, *J* = 4.2, 2.0 Hz, 3H). ^13^C{^1^H} NMR (101 MHz, CDCl_3_) δ 202.61, 137.59, 136.57, 133.89, 127.62, 121.91, 119.75, 118.36, 117.40, 111.17, 104.38, 55.87, 54.99, 50.71, 33.01, 29.85, 27.25, 26.89, 12.79.

#### (±)-*N*-Methylvellosimine (**2**).^4d^

Following general procedure II using **11b** as starting material. Purification by flash column chromatography (CHCl_3_:MeOH 50:1 → 30:1 + 1% TEA) afforded (±)-**2** (7.0 mg, 28% yield) as an off-white solid. HRMS (ESI^+^) calcd. for C_20_H_23_N_2_O ([M + H]^+^) 307.1810, found 307.1810. ^1^H NMR (400 MHz, CDCl_3_) δ 9.64 (s, 1H), 7.47 (d, *J* = 7.7 Hz, 1H), 7.30 (d, *J* = 8.1 Hz, 1H), 7.20 (ddd, *J* = 8.2, 7.0, 1.2 Hz, 1H), 7.10 (td, *J* = 7.4, 1.1 Hz, 1H), 5.36 (q, *J* = 6.7 Hz, 1H), 4.27 (dd, *J* = 9.8, 2.8 Hz, 1H), 3.65 (s, 3H), 3.64–3.58 (m, 3H), 3.23–3.19 (m, 1H), 3.15 (dd, *J* = 15.5, 5.2 Hz, 1H), 2.61 (d, *J* = 15.6, 1H), 2.49 (d, *J* = 7.5 Hz, 1H), 2.24–2.08 (m, 1H), 1.76 (dd, *J* = 12.6, 3.3 Hz, 1H), 1.62 (dt, *J* = 7.0, 2.1 Hz, 3H). ^13^C{^1^H} NMR (101 MHz, CDCl_3_) δ 202.90, 139.33, 137.54, 134.48, 127.35, 121.28, 119.19, 118.39, 117.17, 108.96, 103.32, 56.30, 55.04, 50.72, 49.56, 32.56, 29.54, 27.41, 26.75, 12.81.

#### (±)-10-Methoxyvellosimine (**3**).^4d^

Following general procedure II using **11c** as starting material. Purification by flash column chromatography (CHCl_3_:MeOH 50:1 → 30:1 → 20:1 + 1% TEA) afforded (±)-**3** (22 mg, 68% yield) as a white solid. HRMS (ESI^+^) calcd. For C_20_H_23_N_2_O_2_ ([M + H]^+^) 323.1759, found 323.1752. ^1^H NMR (400 MHz, CDCl_3_) δ 9.64 (d, *J* = 1.2 Hz, 1H), 7.83 (s, 1H), 7.17 (s, 1H), 6.91 (d, *J* = 2.5 Hz, 1H), 6.80 (dd, *J* = 8.7, 2.5 Hz, 1H), 5.34 (d, *J* = 7.2 Hz, 1H), 4.11 (d, *J* = 10.1 Hz, 1H), 3.84 (s, 3H), 3.66–3.54 (m, 3H), 3.26–3.16 (m, 1H), 3.16–3.05 (m, 1H), 2.59–2.50 (m, 1H), 2.05 (dd, *J* = 12.7, 10.2 Hz, 1H), 1.80 (dt, *J* = 12.6, 3.3 Hz, 2H), 1.61 (dt, *J* = 7.4, 2.3 Hz, 3H). ^13^C{^1^H} NMR (101 MHz, CDCl_3_) δ 202.95, 154.34, 138.82, 134.49, 131.54, 128.19, 117.09, 111.72, 111.59, 104.38, 100.64, 70.73, 56.05, 55.11, 50.56, 33.21, 29.85, 27.40, 26.99, 12.78.

#### (−)-Vellosimine, (−)-**1**).^4d^

Product obtained using (−)-(*1R,2R,5R,6R*)-9-benzyl-9-azabicyclo[3.3.1]nonane-2,6-diol as a starting material in an analogous way to racemic vellosimine. (−)-Vellosimine was obtained as an off-white solid. [α]^D^_20_ = −42° (*c* = 0.93).^4g^ HRMS (ESI+) calcd. for C_19_H_21_N_2_O ([M + H]^+^) 293.1653, found 293.1647. ^1^H and ^13^C NMR spectra were identical to those of racemic material.

## Data Availability

The data underlying this study are available in the published article and its online Supporting Information.

## Supporting Information Available

^1^H and ^13^C NMR spectra of new and known compounds (PDF)
