# Supplementary material for: A Short Synthesis of Vellosimine and Its Derivatives
Source: J Org Chem. 2023 Jun 2;88(13):9569–73. doi: 10.1021/acs.joc.3c00905 (PMC11008781; doi:10.1021/acs.joc.3c00905)
Supplement: Supplementary file 1 — jo3c00905_si_001.pdf [file jo3c00905_si_001.pdf]

A Short Synthesis of Vellosimine and Its Derivatives

Barbara Chatinovska, Rokas Gegevičius, Edvinas Orentas*

Department of Organic Chemistry, Institute of Chemistry, Vilnius University, Naugarduko 24, Vilnius, Lithuania

Correspondence to: edvinas.orentas@chf.vu.lt

SUPPORTING INFORMATION

Copies of NMR spectra

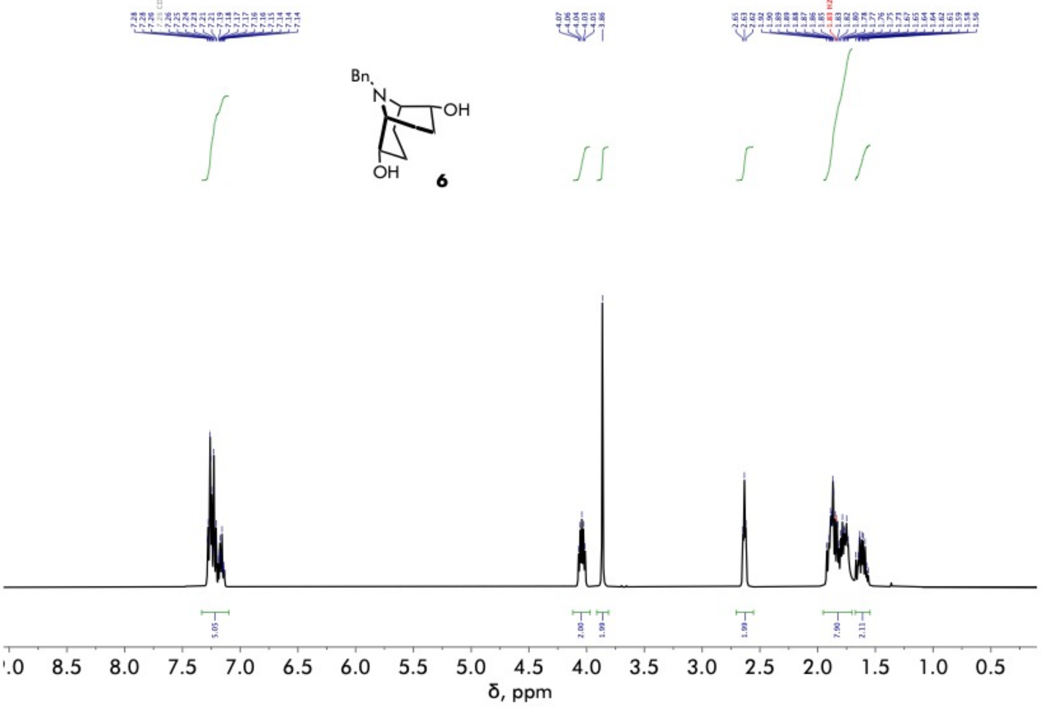

Figure S1. ^{1}H NMR (400 MHz, $CDCl_3$) spectrum of **6**.

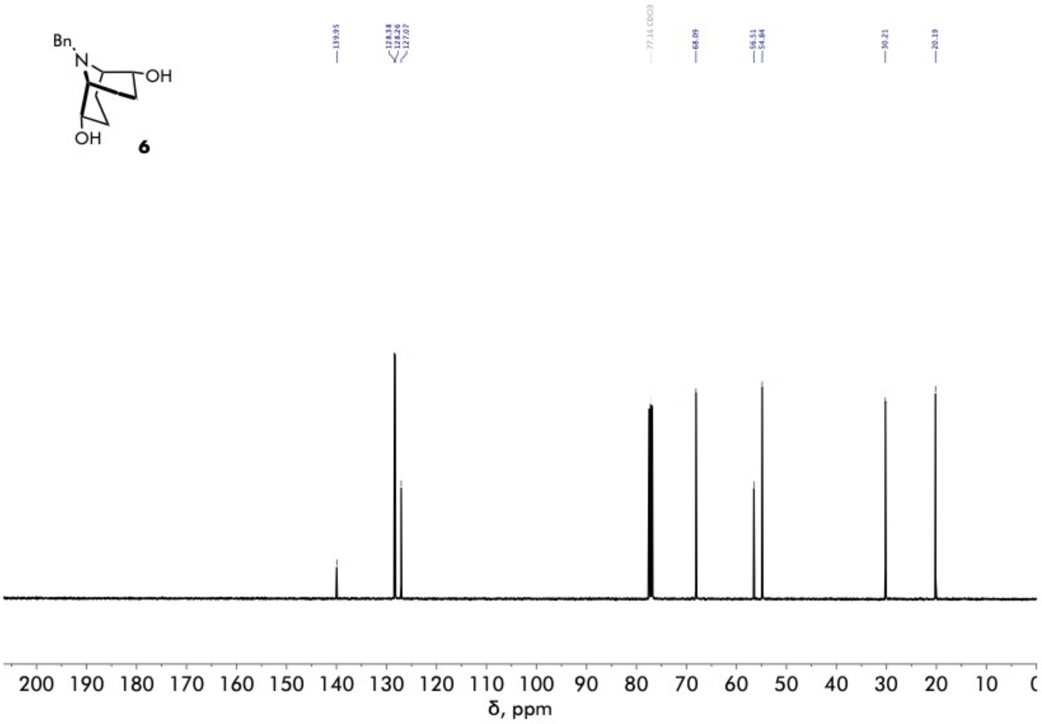

Figure S2. $^{13}C\{^{1}H\}$ NMR (101 MHz, $CDCl_3$) spectrum of **6**.

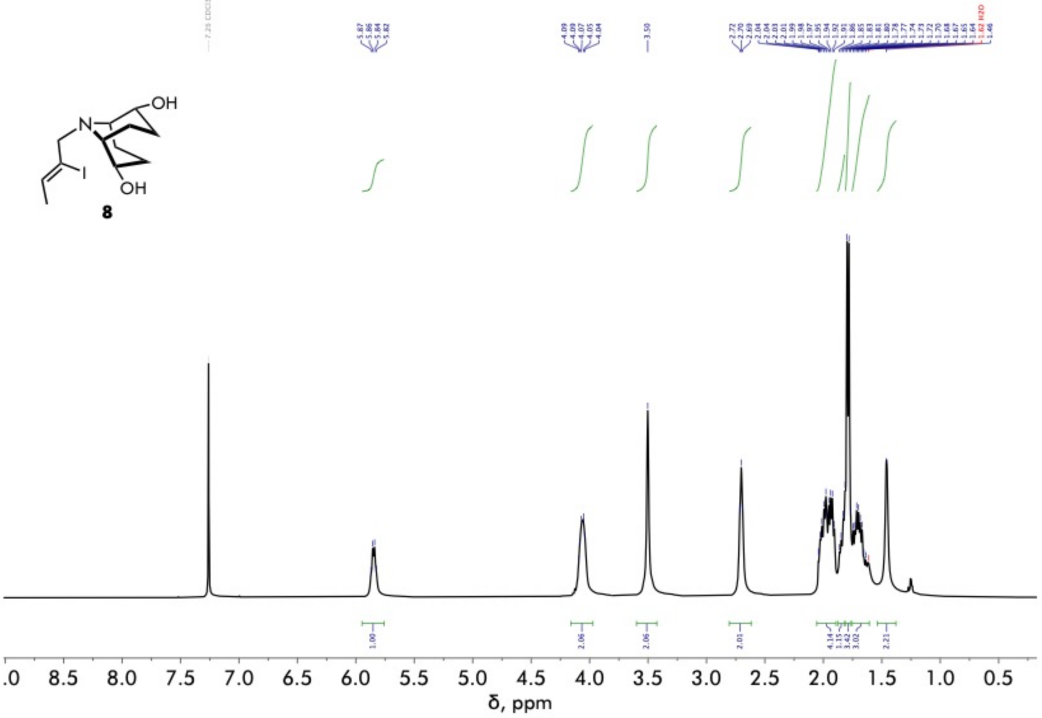

Figure S3. ^{1}H NMR (400 MHz, $CDCl_3$) spectrum of **8**.

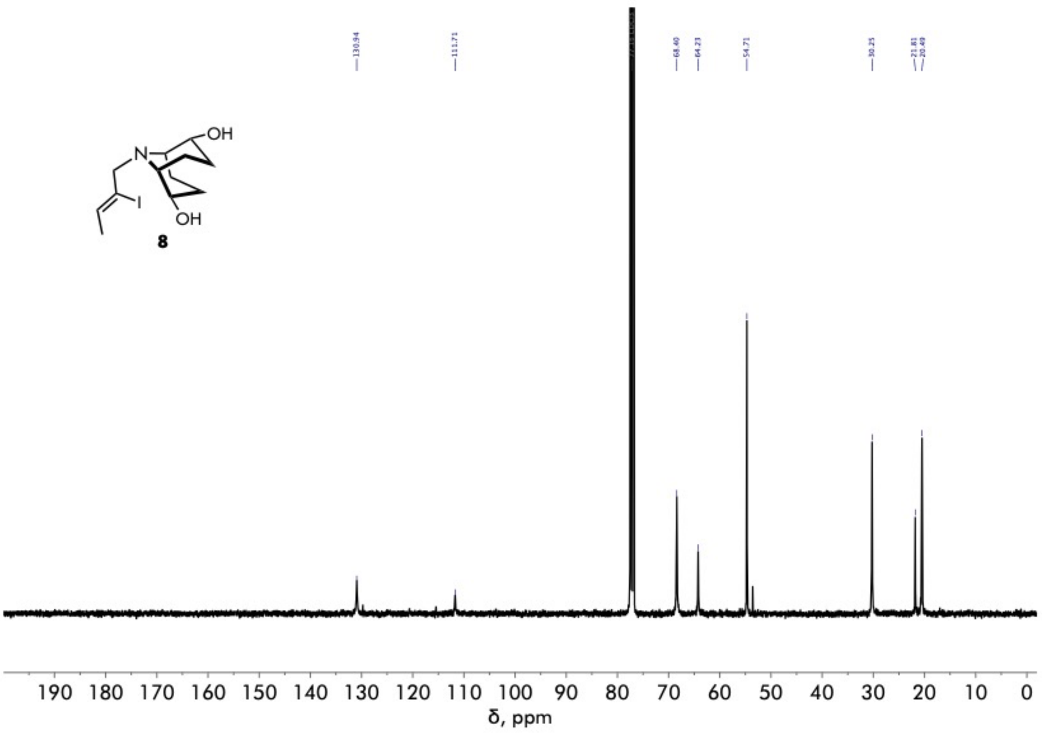

Figure S4. $^{13}C\{^{1}H\}$ NMR (101 MHz, $CDCl_3$) spectrum of **8**.

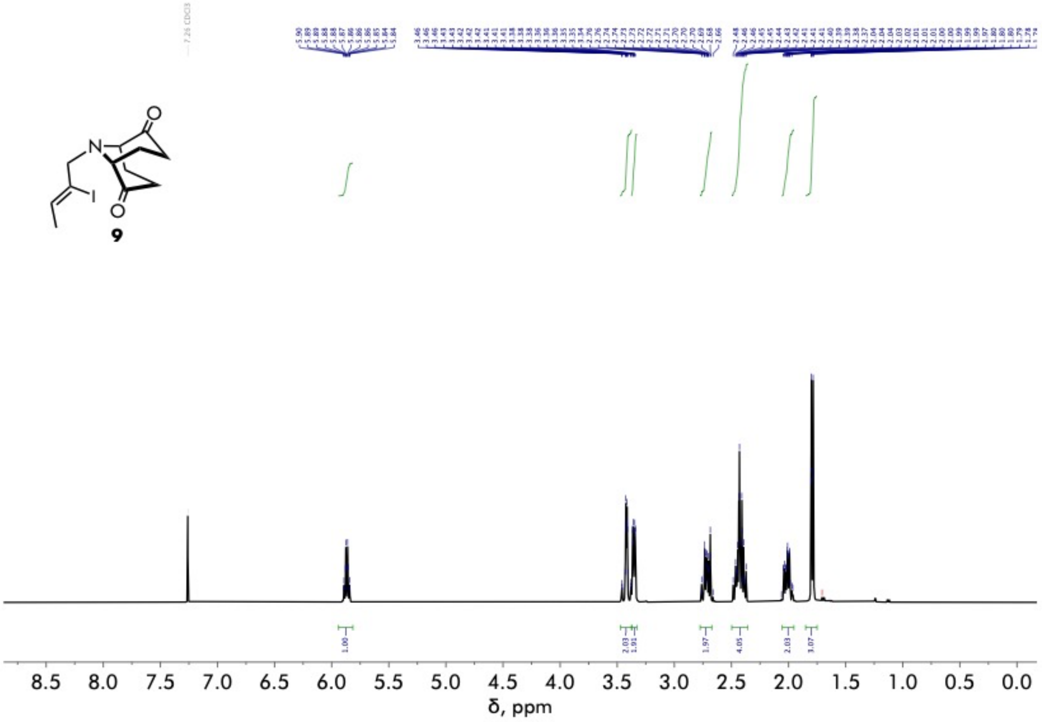

Figure S5. ^{1}H NMR (400 MHz, $CDCl_3$) spectrum of **9**.

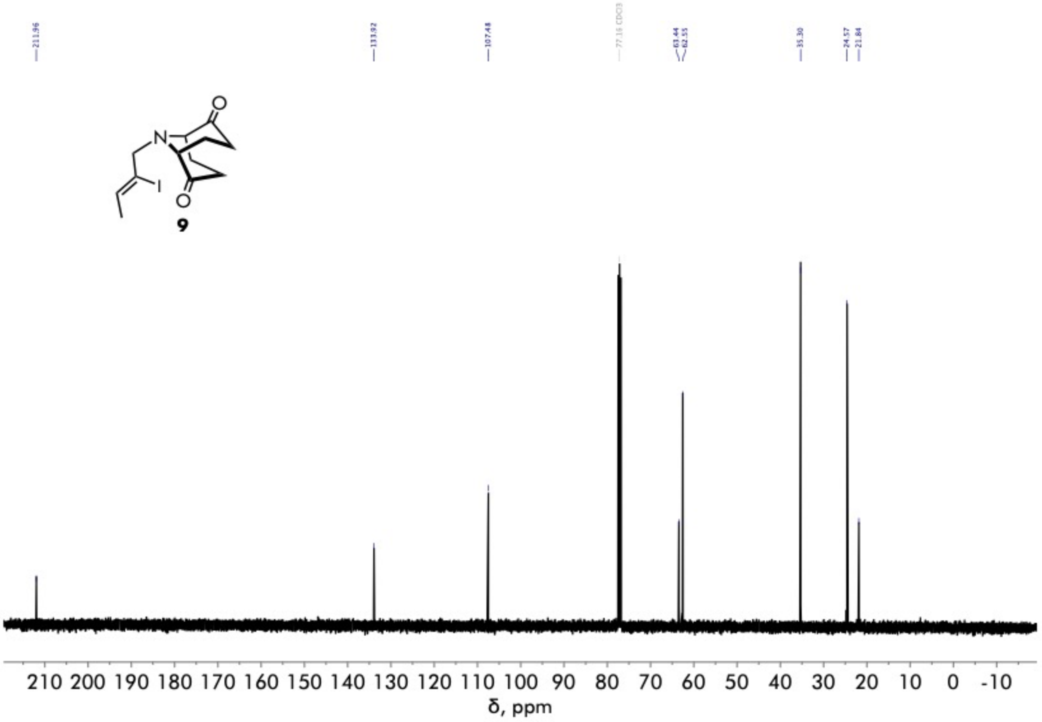

Figure S6. $^{13}C\{^{1}H\}$ NMR (101 MHz, $CDCl_3$) spectrum of **9**.

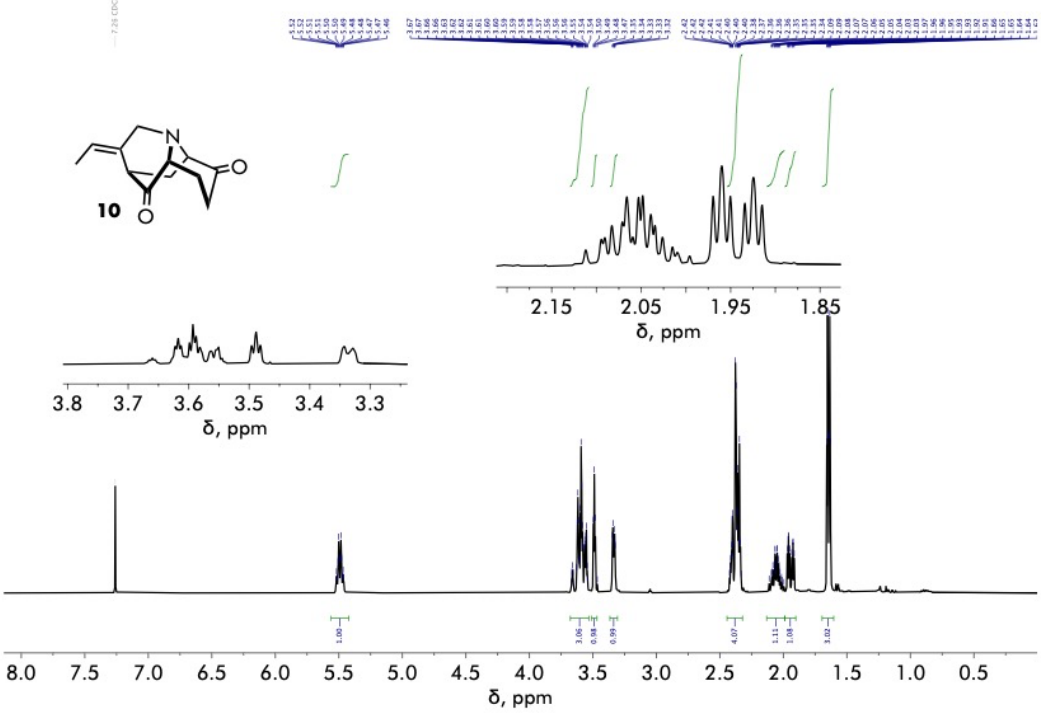

Figure S7. ^{1}H NMR (400 MHz, $CDCl_3$) spectrum of **10**.

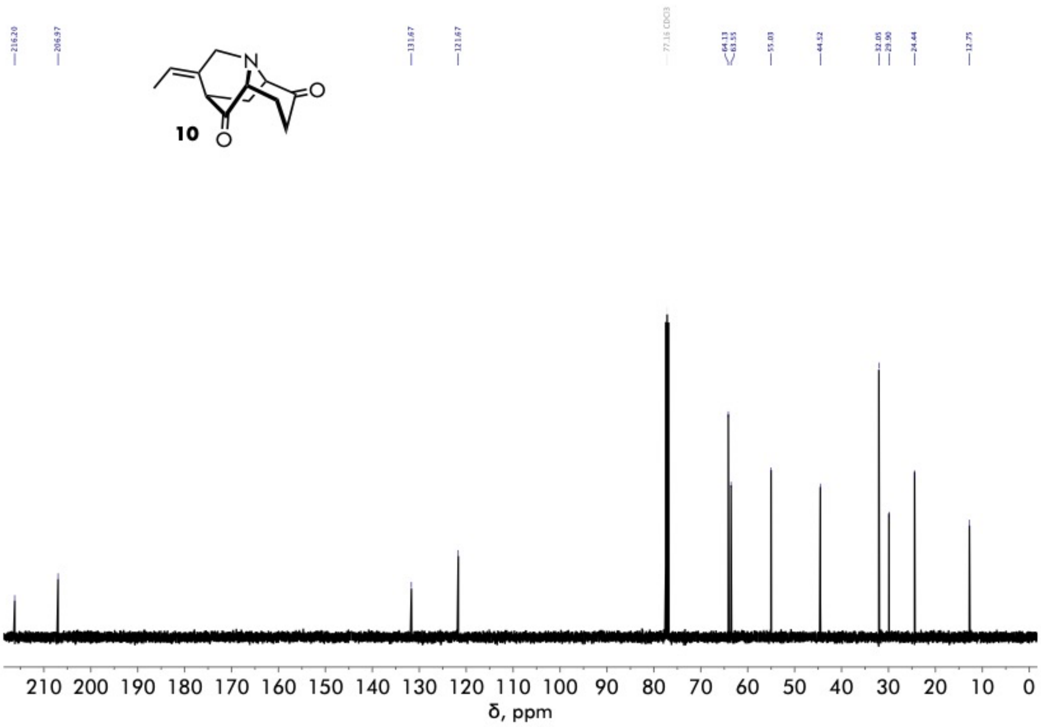

Figure S8. $^{13}C\{^{1}H\}$ NMR (101 MHz, $CDCl_3$) spectrum of **10**.

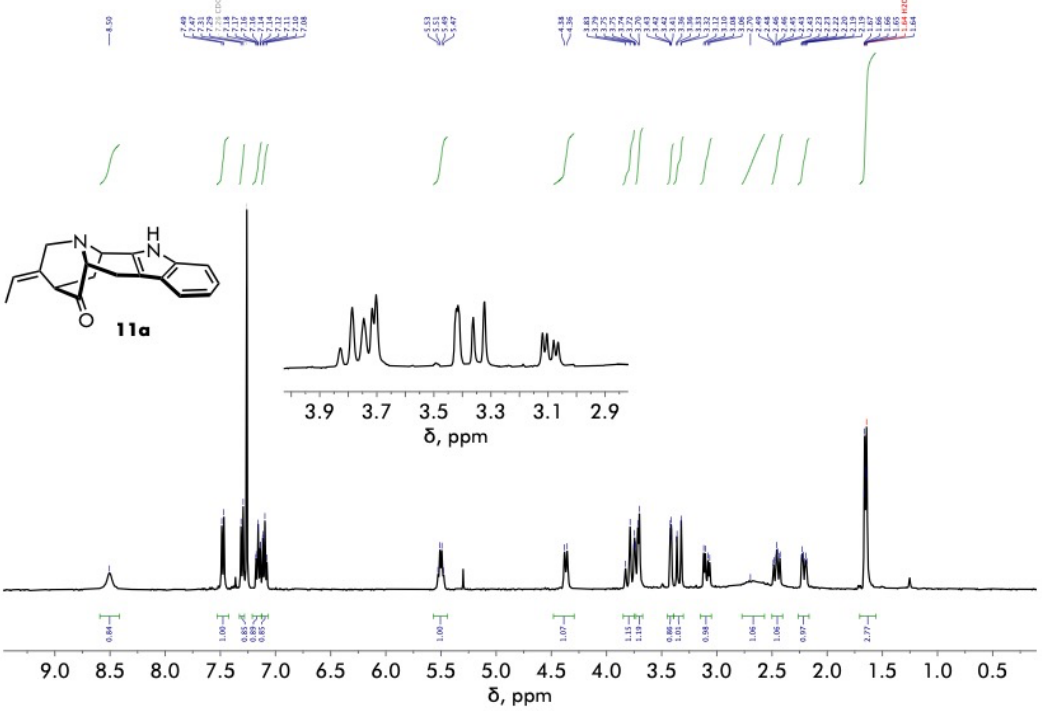

Figure S9. ^{1}H NMR (400 MHz, $CDCl_3$) spectrum of **11a**.

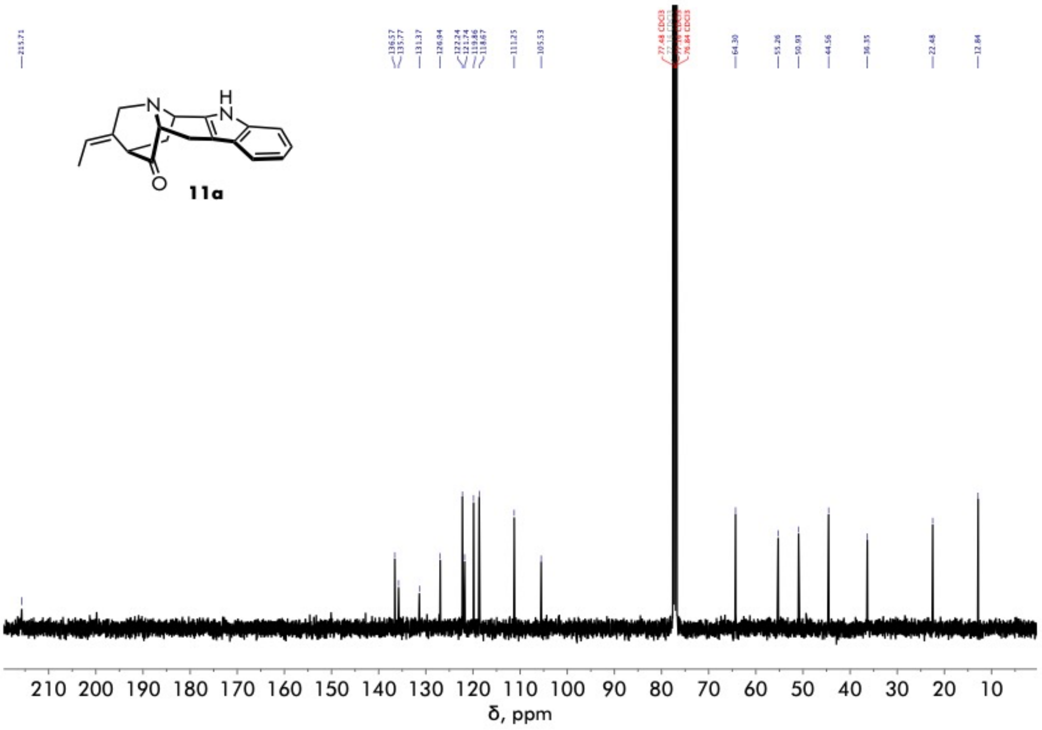

Figure S10. $^{13}C\{^{1}H\}$ NMR (101 MHz, $CDCl_3$) spectrum of **11a**.

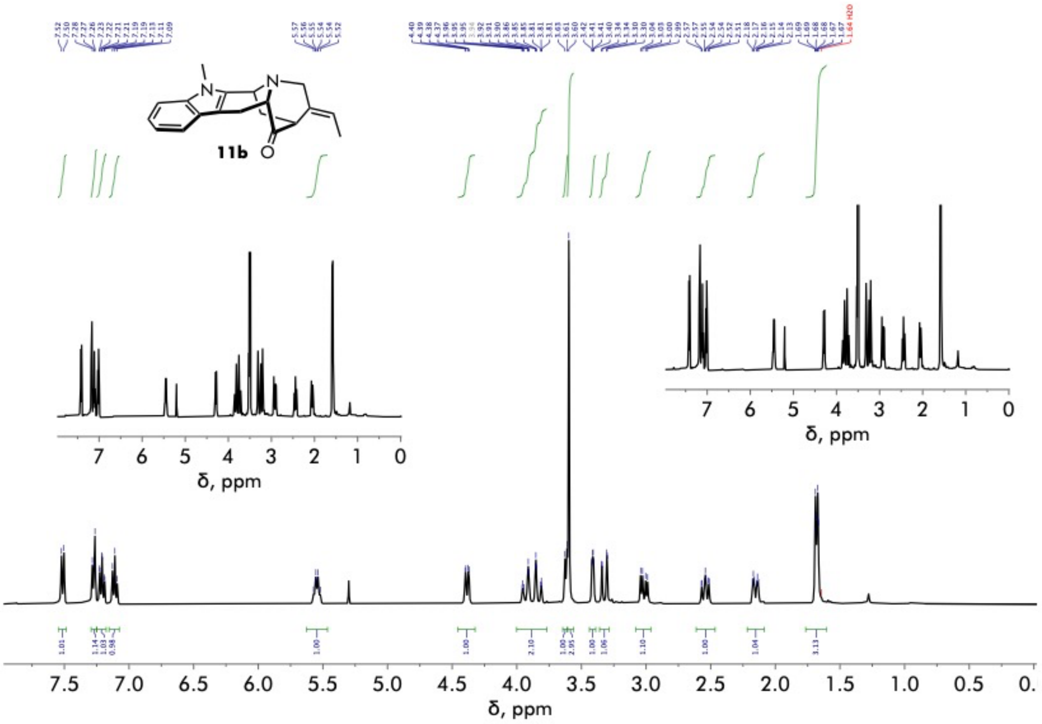

Figure S11. ^{1}H NMR (400 MHz, $CDCl_3$) spectrum of **11b**.

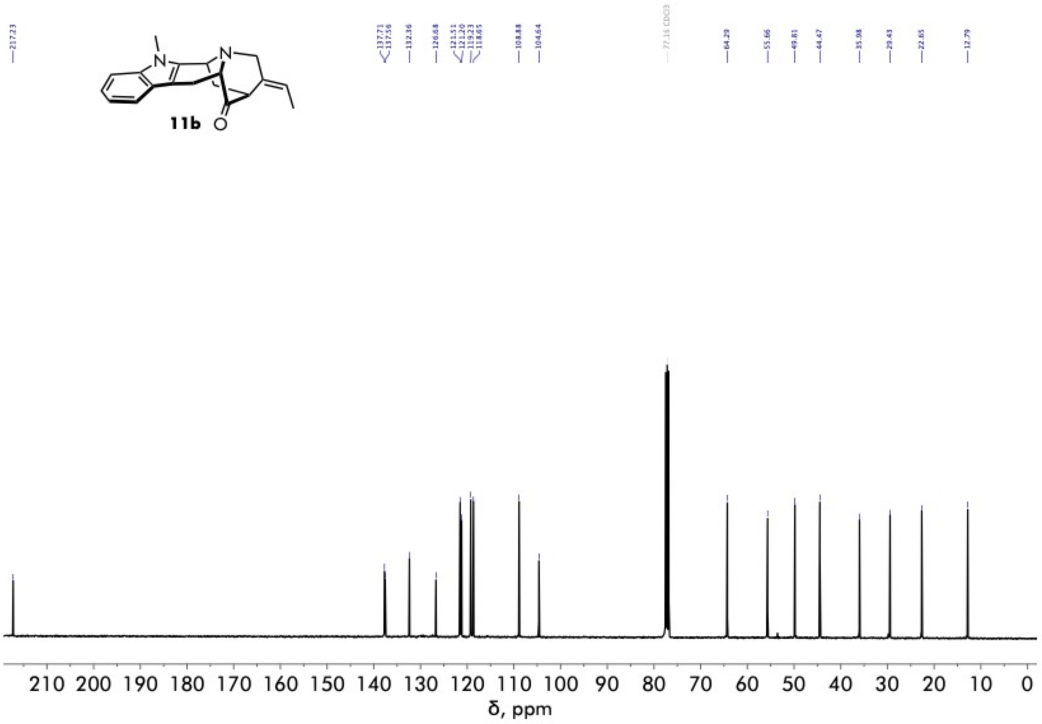

Figure S12. $^{13}C\{^{1}H\}$ NMR (101 MHz, $CDCl_3$) spectrum of **11b**.

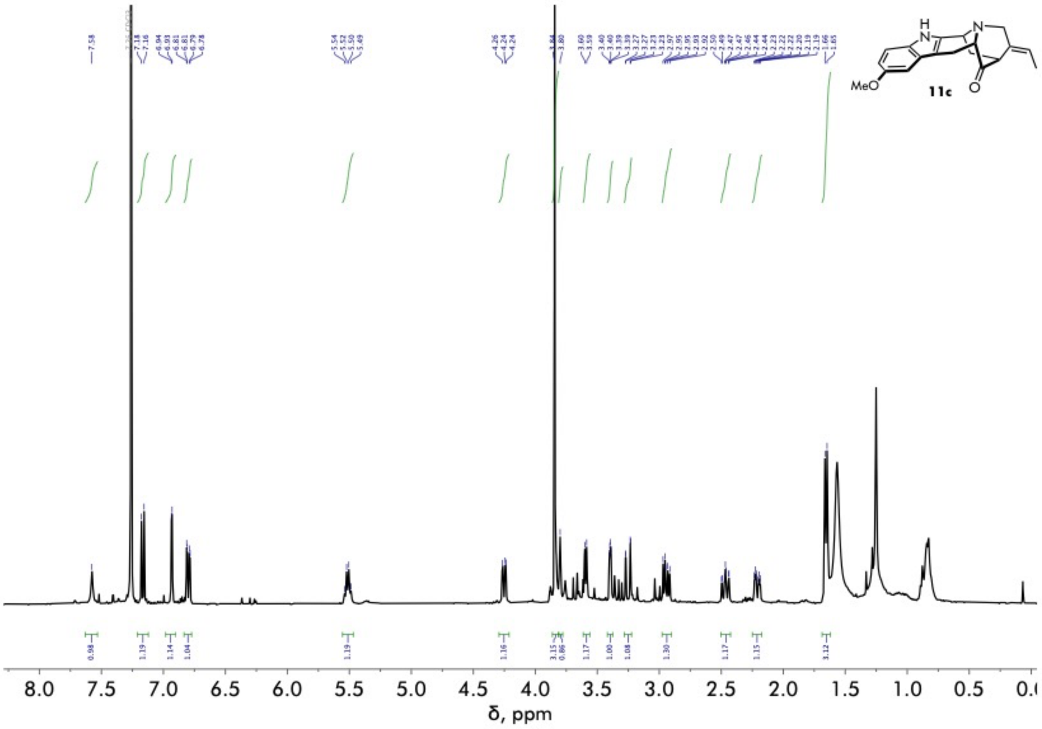

Figure S13. ^{1}H NMR (101 MHz, $CDCl_3$) spectrum of **11c**.

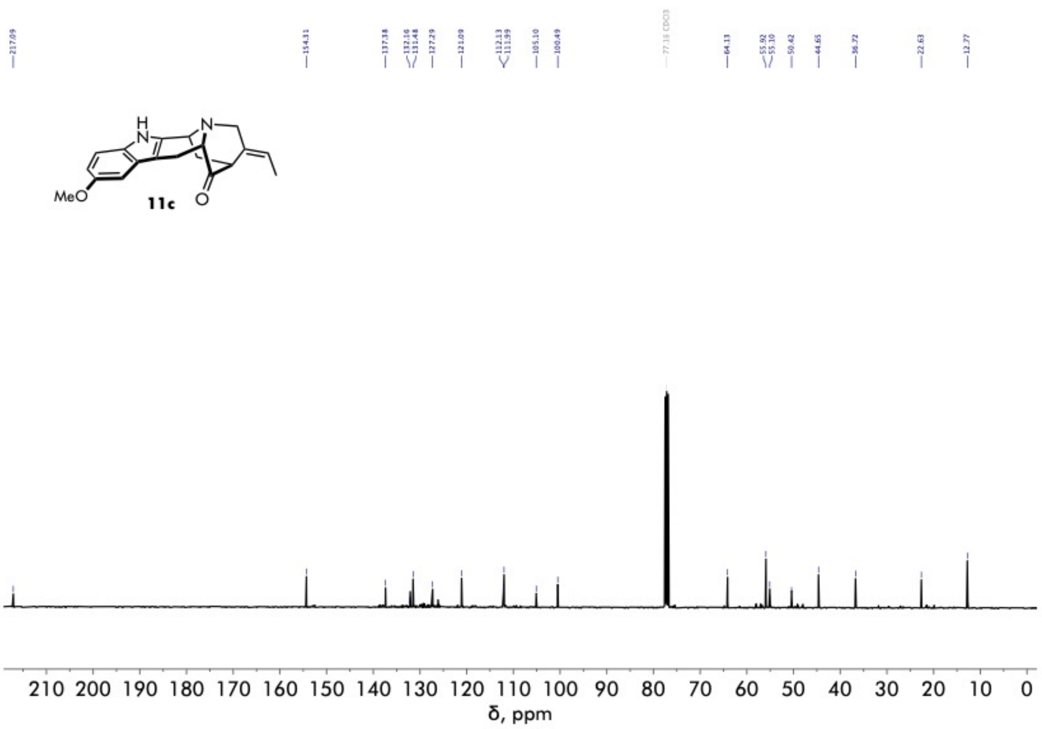

Figure S14. $^{13}C\{^{1}H\}$ NMR (101 MHz, $CDCl_3$) spectrum of **11c**.

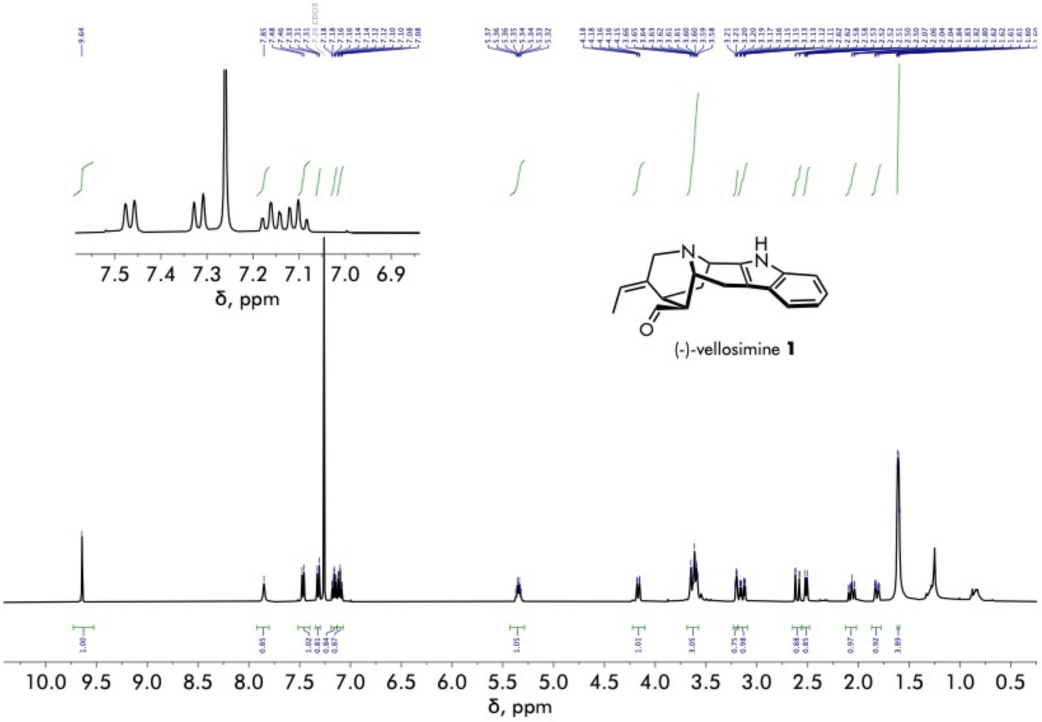

Figure S15. ^{1}H NMR (400 MHz, $CDCl_3$) spectrum of (-)-vellosimine **1**.

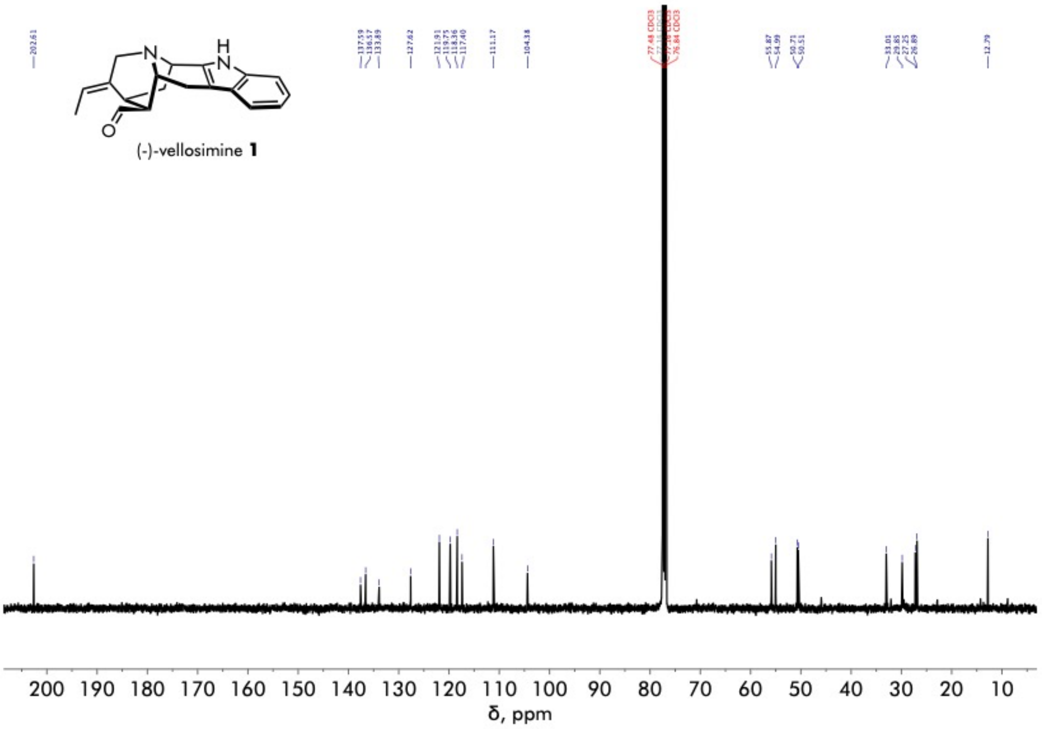

Figure S16. $^{13}C\{^{1}H\}$ NMR (101 MHz, $CDCl_3$) spectrum of (-)-vellosimine **1**.

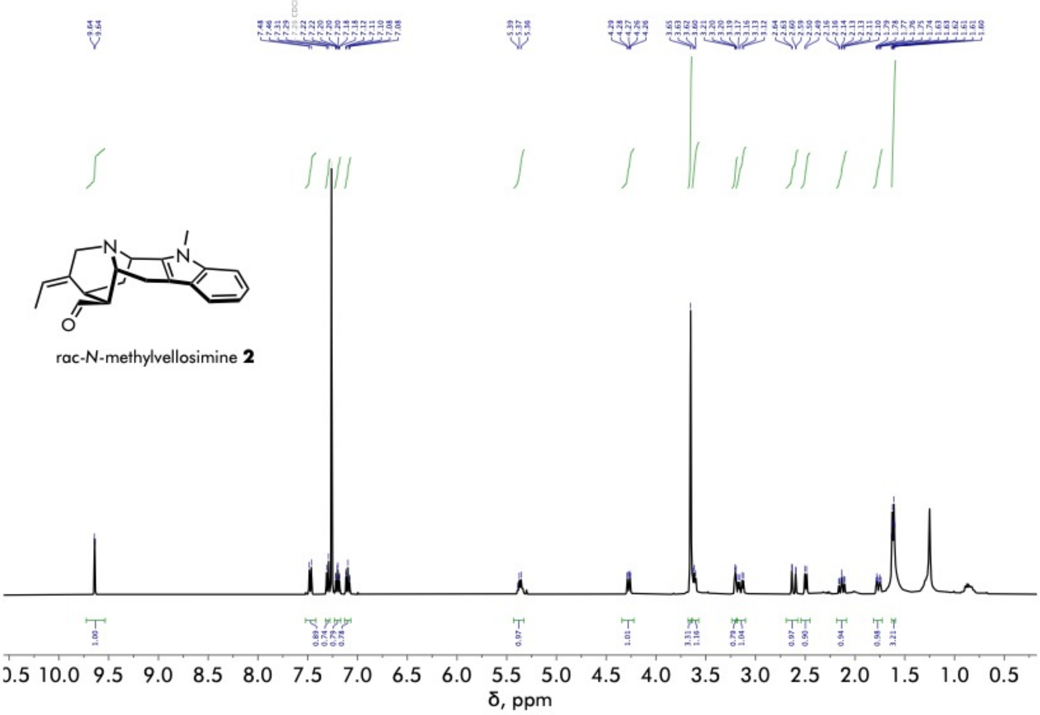

Figure S17. ^{1}H NMR (400 MHz, $CDCl_3$) spectrum of *rac*-*N*-methylvellosimine **2**.

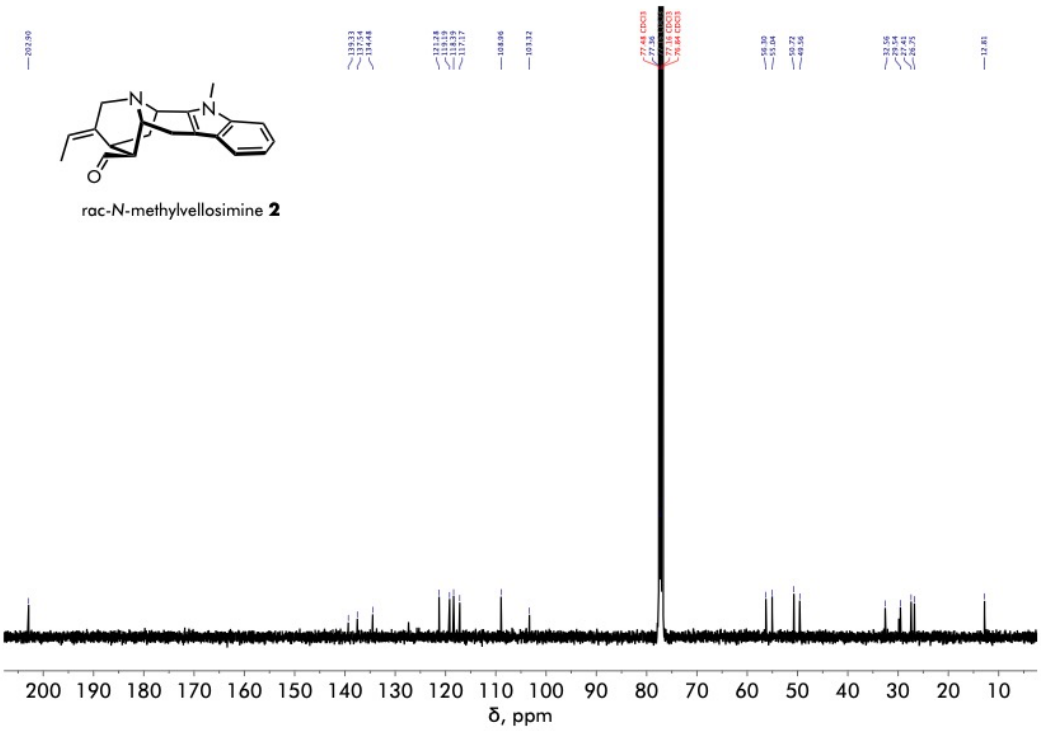

Figure S18. $^{13}C\{^{1}H\}$ NMR (101 MHz, $CDCl_3$) spectrum of *rac*-*N*-methylvellosimine **2**.

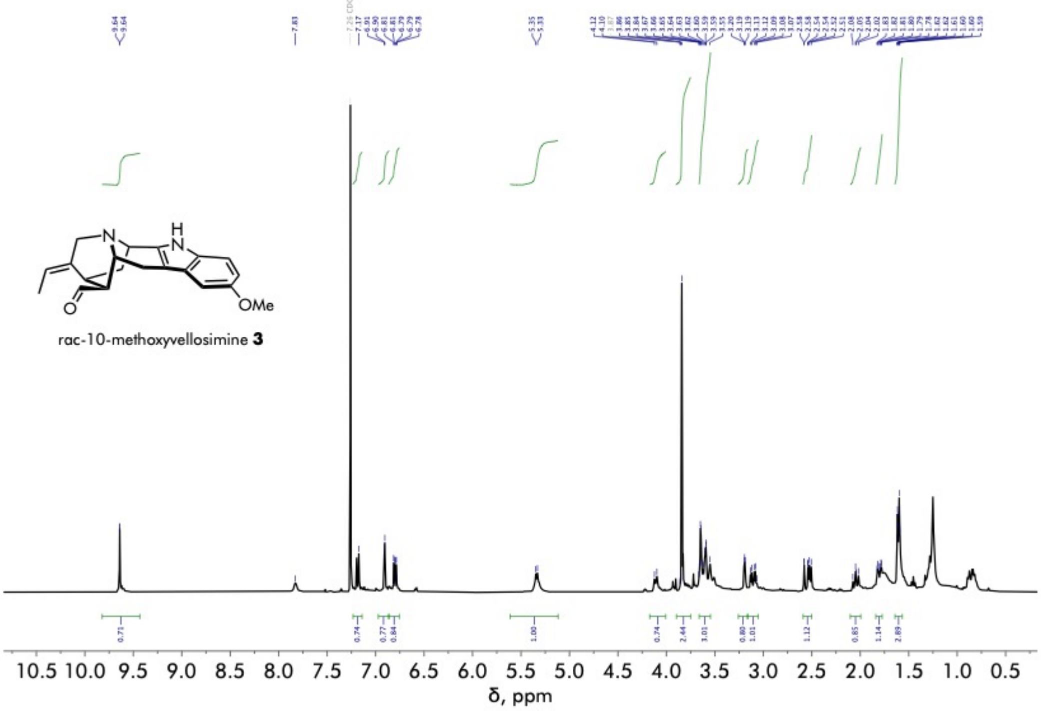

Figure S19. ^{1}H NMR (400 MHz, $CDCl_3$) spectrum of *rac*-10-methoxyvellosimine **3**.

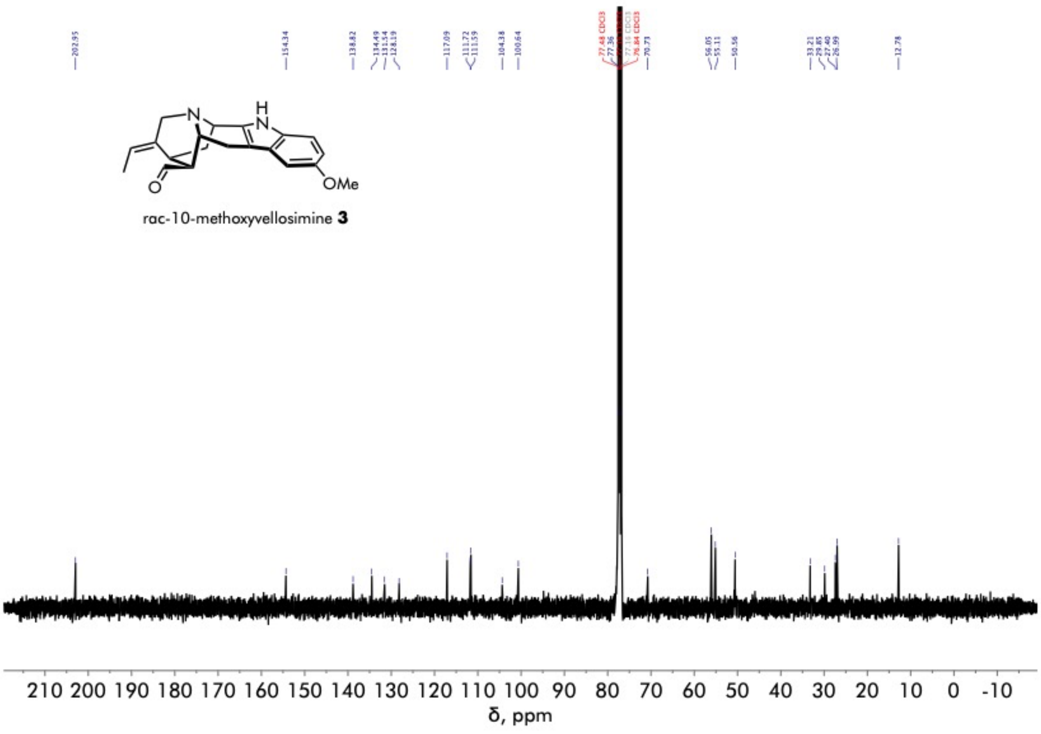

Figure S20. $^{13}C\{^{1}H\}$ NMR (101 MHz, $CDCl_3$) spectrum of *rac*-10-methoxyvellosimine **3**.
